# Predicting weaning difficulty for planned extubation patients with an artificial neural network

**DOI:** 10.1097/MD.0000000000017392

**Published:** 2019-10-04

**Authors:** Meng Hsuen Hsieh, Meng Ju Hsieh, Ai-Chin Cheng, Chin-Ming Chen, Chia-Chang Hsieh, Chien-Ming Chao, Chih-Cheng Lai, Kuo-Chen Cheng, Willy Chou

**Affiliations:** a Department of Electrical Engineering and Computer Science, University of California, Berkeley, Berkeley, CA; b Department of Medicine, Poznan University of Medical Science, Poznan, Poland; c Department of Medical Sciences Industry, Chang Jung Christian University; d Section of Respiratory Care, Department of Internal Medicine, Chi-Mei Medical Center; e Department of Intensive Care Medicine, Chi Mei Medical Center, Tainan; f China Medical University Children's Hospital, China Medical University, Taichung; g Department of Intensive Care Medicine, Chi Mei Medical Center, Liouying District; h Department of Internal Medicine, Kaohsiung Veterans General Hospital, Tainan Branch, Tainan; i Department of Physical Medicine and Rehabilitation, Chi Mei Medical Center, Chiali; j Department of Recreation and Health-Care Management, Chia Nan University of Pharmacy and Science, Tainan, Taiwan.

**Keywords:** artificial neural network, planned extubation, prediction weaning difficulty

## Abstract

This study aims to construct a neural network to predict weaning difficulty among planned extubation patients in intensive care units.

This observational cohort study was conducted in eight adult ICUs in a medical center about adult patients experiencing planned extubation.

The data of 3602 patients with planned extubation in ICUs of Chi-Mei Medical Center (from Dec. 2009 through Dec. 2011) was used to train and test an artificial neural network (ANN) model. The input features contain 47 clinical risk factors and the outputs are classified into three categories: simple, difficult, and prolonged weaning. A deep ANN model with four hidden layers of 30 neurons each was developed. The accuracy is 0.769 and the area under receiver operating characteristic curve for simple weaning, prolonged weaning, and difficult weaning are 0.910, 0.849, and 0.942 respectively.

The results revealed that the ANN model achieved a good performance in prediction the weaning difficulty in planned extubation patients. Such a model will be helpful for predicting ICU patients’ successful planned extubation.

## Abstract 2

Supplemental Digital Content is available in the text

## 1 Introduction

Endotracheal intubation is a process commonly used in intensive care unit (ICU) patients. On average, 39% of ICU patients require endotracheal intubation with ventilatory support.^[1]^ Though required, prolonged ventilatory support can increase the risk of certain complications, such as ventilation-associated pneumonia.^[2]^ The extubation of ventilated patients as early as possible is therefore desired through weaning.^[3]^ Weaning is the process of gradually removing ventilatory support in a patient by the process of extubation.^[4]^ The appropriate time to start the weaning process is determined by clinicians to avoid prolonged ventilatory support.^[5]^ Therefore, weaning profiles and extubation predictions are very important in assessing a patient undergoing endotracheal intubation.

In 2005, during the international consensus conference on weaning from mechanical ventilation, a patient classification system according to the weaning process was proposed. According to the duration of weaning and the number of spontaneous breathing trials (SBTs) preceding successful extubation, patients are classified into three groups: simple, difficult, and prolonged weaning.^[4]^ This weaning classification has been evaluated in clinical practice.^[6,7]^ In the studies, the prolonged weaning group was associated with increased mortality in the ICU.

In recent years, outcome prediction models using artificial neural network (ANN) and multivariable logistic regression analysis have been developed in many areas of health care research.^[8]^ There is a growing amount of publications regarding the use of machine learning algorithms in ICU subjects, particularly in the prediction of sepsis as well.^[9,10,11]^ ANNs are computer-based algorithms that mimic the habits and structures of neurons and can derive outcomes based on input data. With a clear classification system for patient weaning profiles, the aim of our study is to utilize ANNs to categorize patients into them to predict their individual weaning difficulty.

## 2 Materials and methods

### 2.1 Study design and setting

This study was a retrospective analysis using machine learning method based on the data collected based on a previous prospective study, which was conducted in eight adult ICUs of Chi-Mei Medical Center from December 2009 through December 2011. This is a 1288-bed tertiary medical center with 96 ICU beds: 48 medical ICU beds, 9 cardiac beds, and 39 surgical beds for adults. Every year, more than 5000 patients are admitted to the ICU in average. The ICU is covered by intensivists, senior residents, nurses, respiratory therapists, dietitians, physical therapists, and clinical pharmacists. The workload is the same in every shift and patient-to-nursing staff ratios of 2:1. There were no differences in nursing experience by shift. Each respiratory therapist was responsible for fewer than 10 patients at the same time on every shift. The ICU team made rounds at least once daily, and the physician decided the timing of initiating weaning process.

During the study period, a total of 3602 patients experiencing planned extubation were enrolled in this study. According to the weaning process classifications, all patients were separated into three groups: simple, difficult, and prolonged weaning. The definition of simple weaning is a successful extubation after the first SBT; an SBT trial is defined as a low-pressure support with ≤8 cm H_2_O, or a T-piece trial.^[4]^ Difficult weaning is defined by a successful extubation after two or three SBTs, or a successful extubation within seven days of the first SBT. Prolonged weaning is classified by patients not weaned after more than three SBTs, or a weaning process greater than seven days. If the patients displayed unstable hemodynamics or desaturation during SBT trial, we would stop the trial. Either adjusting a higher support level or shifting to control mode would be performed for failed SBT. All patients’ demographic and clinical information, laboratory results, comorbidities, severity scores, mortality, and lengths of stays for both ICU and hospital were collected for analysis. The data were retrospectively collected before planned extubation after passed SBT and then analyzed. Therefore, informed consent was specifically waived and the study was approved by the Institutional Review Board of Chi Mei Medical Center (IRB: 10706-009).

#### 2.1.1 Constructing the training data

All features are extracted from the original dataset. After normalizing and cleaning the data, there are 47 input features and three outputs, each representing a prediction of simple, difficult, and prolonged weaning. The 47 input features include subject age, gender, scoring systems as Acute Physiology and Chronic Health Evaluation II (APACHE-II), Therapeutic Intervention Scoring System (TISS) and Glasgow Coma Scales, comorbidities, etiology of intubation and respiratory failure, pre-extubation parameters, weaning methods and parameters, and pre-extubation data. The basis features are listed in Table 1 in the Supplemental Digital Content. The data is then split into training and test sets at an approximately 9:1 ratio.^[12]^Table 1 shows the data allocation between the test and train sets.

**Table 1:**
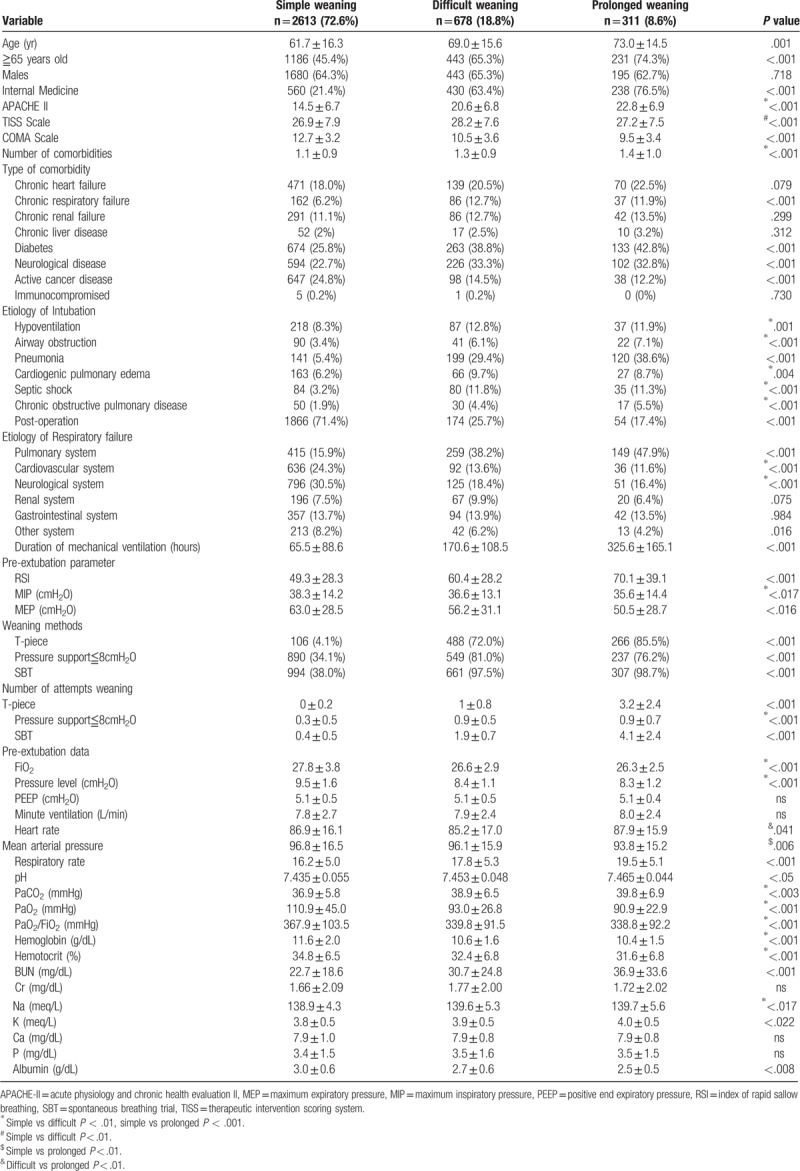
Baseline characteristics of the 3602 patients who started weaning, stratified by weaning category.

#### 2.1.2 Algorithm and training

We used a multilayer perceptron deep neural network to train the data. To select the hyperparameters, optimizers, and loss function with the best performance, *k*-fold cross-validation with a *k* value of 10 is used over 10 epochs.^[12]^

After the model selection process of comparing the performance of different models with *k*-fold cross-validation, the best-performing model consisted of one input layer of 47 dimensions, 4 hidden layers of 30 dimensions each, and an output layer of 3 dimensions. The network was trained using stochastic gradient descent and optimized using Adam with Nesterov Momentum.^[13]^ The input and hidden layers used the Scaled Exponential Linear Unit (SeLU) activation function, while the output layer used the Softmax activation function.^[14]^ Dropout of 20% was applied at the input layer and 50% at the output layer for regularization.^[15]^ The neuron weights were initialized using normalized He initialization.^[16]^ Since the ANN aims to solve a multi-class classification problem, the categorical cross-entropy function was used as the loss function. The model generates a probability for each category, and the patient is assigned to the category with the highest probability.

The software was implemented using Python (version 3.7.0) with the scikit-learn library (version 0.19.1).^[17]^ The ANN model was created and trained with the Tensorflow framework (version 1.9.0).^[18]^

#### 2.1.3 Statistical analyses

Mean values, standard deviations, and group sizes were used to summarize the results for continuous variables. Kruskal–Wallis ANOVA was used for comparison of continuous variables with Dunn's test for post hoc testing. The Chi-squared test for trends was used to compare categorical variables between the three weaning categories. A *P* value < .05 was considered statistically significant. Statistical analysis of the data was done with SPSS 21.0 for Windows (SPSS, Inc., IL).

The ANN performance was measured using the area under Receiving Operating Characteristic (ROC) curve. The area under ROC curve (AUROC) of the neural network was compared against the AUC of variables that had a significant difference in terms of outcomes. The AUC was also compared against the ideal value of one.^[19]^

## 3 Results

### 3.1 Results of clinical data

Of the 3602 patients included in the study, 50.9% were male and 49.1% were female. Patients were classified according to weaning classification: 2613 patients (72.6%) as simple weaning, 678 patients (18.8%) as difficult weaning, and 311 (8.6%) as prolonged weaning. The mean age of simple weaning group is 61.7 years, the mean age of difficult weaning group is 69.0 years, while the mean age of prolonged weaning group is 73.0 years. The mean APACHE II score in difficult group is 20.6 and in prolonged group is 22.8, and both are significantly higher than that of simple weaning group, which is 14.5 (*P* < .001). The most common comorbidity is diabetes in the prolonged weaning group (42.8%) and in the difficult weaning group (38.8%). See Table 1 in the Supplemental Digital Content for the full demographic and clinical characteristics ICU patients with planned extubation. The rate of extubation failure is 5.1% (185/3602).

### 3.2 Results of ANN

**Figure 1:**
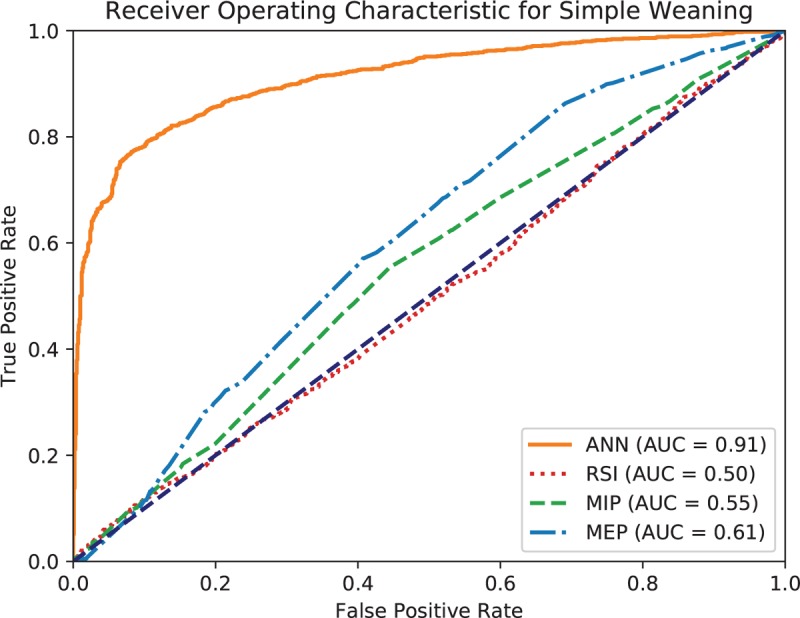
Receiving operating characteristic (ROC) curve of artificial neural network (ANN), rapid shallow breathing (RSI), maximum expiratory pressure (MEP), and maximum inspiratory pressure (MIP) for simple weaning.

**Figure 2:**
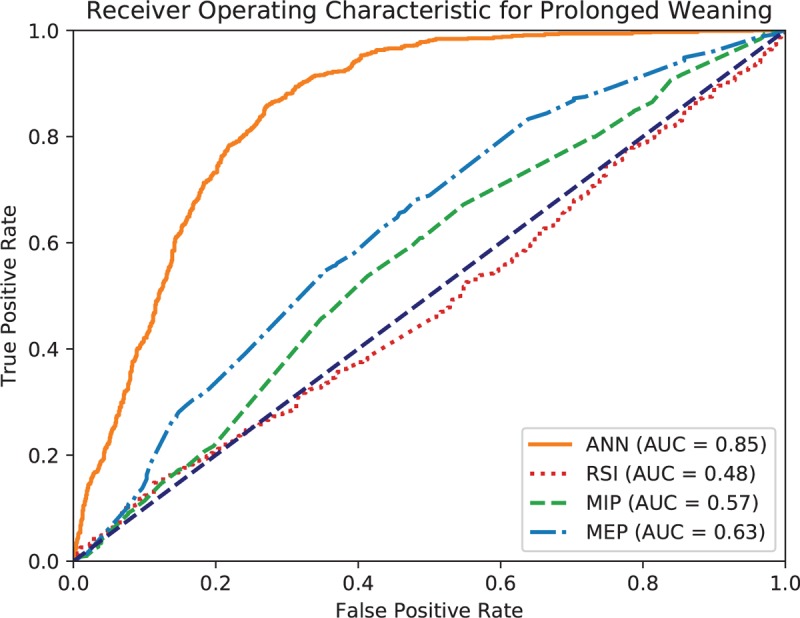
Receiving operating characteristic (ROC) curve of artificial neural network (ANN), rapid shallow breathing (RSI), maximum expiratory pressure (MEP), and maximum inspiratory pressure (MIP) for prolonged weaning.

**Figure 3:**
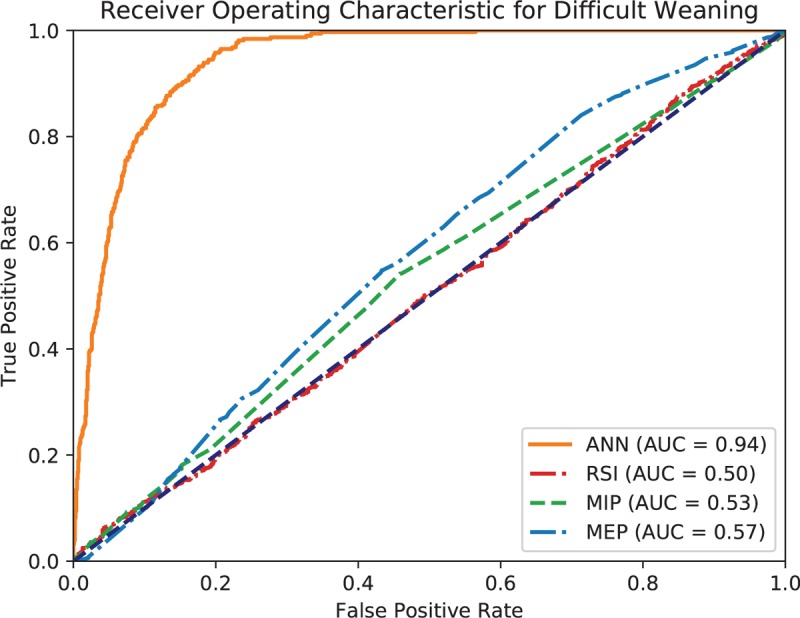
Receiving operating characteristic (ROC) curve of artificial neural network (ANN), rapid shallow breathing (RSI), maximum expiratory pressure (MEP), and maximum inspiratory pressure (MIP) for difficult weaning.

The accuracy of the ANN model is 0.769, and weighted *k*-fold cross-validation accuracy is 0.604. Figures 1 to 3 show the ROC curve of the artificial neural network, rapid shallow-breathing index (RSI), maximum expiratory pressure (MEP), and maximum inspiratory pressure (MIP) on all patient data for simple, prolonged, and difficult weaning respectively. Tables 2 and 3 show the AUROCs of all 3 weaning types for the ANN model and control predictors respectively. The AUROCs were calculated across all data.

**Table 2:**
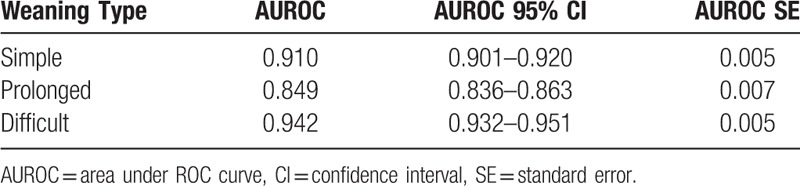
Area under ROC curve of ANN model in predicting the type of weaning across all data.

**Table 3:**
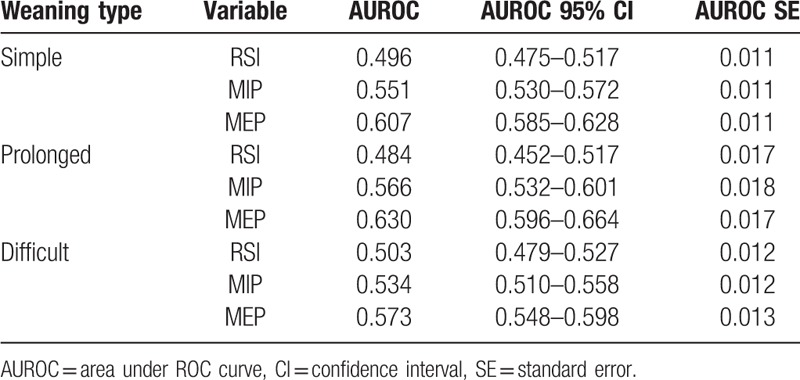
Area under ROC curve of RSI, MSI, and MEP in predicting the type of weaning across all data.

## 4 Discussion

In this study, we demonstrated that a neural network model can be a good predictor for determining weaning classifications. As extubation failure remains prevalent in clinical practice, with reintubation rates reporting up to 19%,^[20]^ it is important to determine patients’ weaning profiles. In common clinical practices, the extubation decision is based on a comprehensive assessment that considers a patient's clinical condition, arterial blood gases results, ventilator settings and weaning profiles.^[5]^ Despite this comprehensive assessment, extubation decisions carries risks of misjudgments that can be fatal. The model created in this study can aid in making decisions for patient extubation with laboratory data.

Additionally, the usage of ANNs in mortality prediction has been recorded since 2006, with its effectiveness noted. The ANN used in the 2006 study concluded that ANN mortality prediction outperformed traditional methods of prognosis assessment.^[21]^ With improved computing power since 2006, ANN models have significantly improved; as such, the ANN used in this study corroborates with the findings and can successfully categorize data into the preset categories for weaning prediction. We found that the collective predictive performance of ANN is better than several commonly used individual parameters in extubation assessment – Index of RSI, MIP, and MEP. This is consistent with a previous study^[8]^ where the proposed ANN model had better discrimination than existing predictors, such as the RSI and MIP, in predicting successful extubation. This shows the widespread potential for ANN models in multiple scenarios wherever categorization and prediction is necessary.

Moreover, previous studies attempted to find appropriate predictors of weaning difficulty and presented different findings. These findings include older age, lower mean arterial pressure,^[22,23]^ arterial carbon dioxide tension (PaCO_2_) under SBT and heart rate increase,^[7]^ lower BUN,^[24]^ MIP and PaCO_2_,^[25]^ as well as the incorporation of respiratory rate, RSI, MIP, and APACHE II scores^[26]^ to aid in the prediction of weaning difficulty. In this study, all the aforementioned factors were included in the ANN model. Thus, the ANN model developed in this study can provide an accurate prediction based on the comprehensive information.

The usage of ANNs in the prediction of patient outcome in ventilator weaning has also been documented. However, the lack of patient data is noted to be a limitation of the studies. Arizmendi et al ran an ANN with 149 patients for the extubation process with a successful predictive capability but failed to categorize patient diagnosis criteria due to the low number of data points.^[27]^ For our study, using our ANN model with 3602 patient data points, the classification of patient weaning outcomes can be performed before the decision to extubate. The weaning classifications can even be used to further predict mortality.^[7]^ This allows for a tool to aid in considering extubation decisions for physicians that can ultimately prevent otherwise dangerous extubation procedures.

The ANN built in this study uses the opensource TensorFlow framework, which allows for easy reproduction of the study; further studies can be reproduced using differing data for a more comprehensive overview. Besides neural networks, other machine learning models such as Gaussian Naïve Bayes (NB), Decision Trees (DT), Linear Discriminant Analysis (LDA), and Support Vector Machines (SVM) can also be studied in the future.

## 5 Conclusions

Extubation strategies in ventilated ICU patients must be thoroughly planned. Previously, the clinical classifications of patient weaning difficulty are used as a characteristic of a patient, rather than assisting in the formulation of a strategy. The ANN used in this study showed that the patient classification can be accurately predicted before the weaning process. This allows the consideration of a patient weaning difficulty prior to the extubation procedure worthwhile.

## Author contributions

**Conceptualization:** Meng Hsuen Hsieh, Ai-Chin Cheng, Chin-Ming Chen, Chih-Cheng Lai, Willy Chou.

**Data curation:** Ai-Chin Cheng, Chin-Ming Chen, Chien-Ming Chao, Kuo-Chen Cheng.

**Formal analysis:** Meng Hsuen Hsieh, Meng Ju Hsieh, Ai-Chin Cheng, Chin-Ming Chen, Chia-Chang Hsieh, Chien-Ming Chao, Kuo-Chen Cheng, Willy Chou.

**Investigation:** Meng Hsuen Hsieh, Meng Ju Hsieh, Ai-Chin Cheng, Chin-Ming Chen, Chia-Chang Hsieh, Chien-Ming Chao, Chih-Cheng Lai.

**Methodology:** Meng Hsuen Hsieh, Meng Ju Hsieh, Ai-Chin Cheng, Chin-Ming Chen, Chia-Chang Hsieh, Chien-Ming Chao, Chih-Cheng Lai, Willy Chou.

**Project administration:** Ai-Chin Cheng, Chin-Ming Chen, Chien-Ming Chao, Kuo-Chen Cheng.

**Resources:** Meng Hsuen Hsieh, Meng Ju Hsieh, Ai-Chin Cheng, Chin-Ming Chen, Chien-Ming Chao, Kuo-Chen Cheng.

**Software:** Meng Hsuen Hsieh, Meng Ju Hsieh, Chin-Ming Chen, Willy Chou.

**Supervision:** Ai-Chin Cheng, Chin-Ming Chen, Chia-Chang Hsieh, Chien-Ming Chao, Chih-Cheng Lai, Kuo-Chen Cheng, Willy Chou.

**Validation:** Meng Hsuen Hsieh, Meng Ju Hsieh, Willy Chou.

**Visualization:** Meng Hsuen Hsieh, Meng Ju Hsieh.

**Writing – original draft:** Meng Hsuen Hsieh, Meng Ju Hsieh, Chin-Ming Chen, Chia-Chang Hsieh, Chih-Cheng Lai.

**Writing – review & editing:** Meng Hsuen Hsieh, Meng Ju Hsieh, Chin-Ming Chen, Chia-Chang Hsieh, Chih-Cheng Lai, Willy Chou.

## Supplementary Material

Supplemental Digital Content
