# Supplementary material for: Predicting weaning difficulty for planned extubation patients with an artificial neural network
Source: Medicine (Baltimore). 2019 Oct 4;98(40):e17392. doi: 10.1097/MD.0000000000017392 (PMC6783239; doi:10.1097/MD.0000000000017392)
Supplement: Supplemental Digital Content [file medi-98-e17392-s001.doc]

Supplemental Digital Content Table 1. Distribution of Train and Test sets

|  | All patients | Train Set | Test Set |
| --- | --- | --- | --- |
| n | 3602 | 3242 | 360 |

Supplemental Digital Content Table 2. Baseline characteristics of the 3602 patients who started weaning, stratified by weaning category

| **Variable** | **Simple weaning**  **n=2613(72.6%)** | **Difficult weaning**  **n=678(18.8%)** | **Prolonged weaning**  **n=311(8.6%)** | **p value** |
| --- | --- | --- | --- | --- |
| Age (years) | 61.7±16.3 | 69.0 ±15.6 | 73.0±14.5 | 0.001 |
| ≧65 years old | 1186(45.4%) | 443(65.3%) | 231(74.3%) | <0.001 |
| Males | 1680 (64.3%) | 443 (65.3%) | 195 (62.7%) | 0.718 |
| Internal Medicine | 560(21.4%) | 430(63.4%) | 238(76.5%) | <0.001 |
| APACHE II | 14.5±6.7 | 20.6±6.8 | 22.8±6.9 | *<0.001 |
| TISS Scale | 26.9±7.9 | 28.2±7.6 | 27.2±7.5 | ^#^<0.001 |
| COMA Scale | 12.7±3.2 | 10.5±3.6 | 9.5±3.4 | <0.001 |
| Number of comorbidities | 1.1± 0.9 | 1.3±0.9 | 1.4± 1.0 | *<0.001 |
| **Type of comorbidity** |  |  |  |  |
| Chronic heart failure | 471 (18.0%) | 139 (20.5%) | 70 (22.5%) | 0.079 |
| Chronic respiratory failure | 162 (6.2%) | 86 (12.7%) | 37(11.9%) | <0.001 |
| Chronic renal failure | 291 (11.1%) | 86 (12.7%) | 42 (13.5%) | 0.299 |
| Chronic liver disease | 52 (2%) | 17 (2.5%) | 10 (3.2%) | 0.312 |
| Metabolic dysfunction | 674 (25.8%) | 263 (38.8%) | 133 (42.8%) | <0.001 |
| Neurological disease | 594 (22.7%) | 226 (33.3%) | 102 (32.8%) | <0.001 |
| Active cancer disease | 647 (24.8%) | 98 (14.5%) | 38 (12.2%) | <0.001 |
| Immunocompromised | 5(0.2%) | 1(0.2%) | 0(0%) | 0.730 |
| **Etiology of Intubation** |  |  |  |  |
| Hypoventilation | 218 (8.3%) | 87 (12.8%) | 37 (11.9%) | *0.001 |
| Airway obstruction | 90 (3.4%) | 41 (6.1%) | 22 (7.1%) | *<0.001 |
| Pneumonia | 141(5.4%) | 199 (29.4%) | 120 (38.6%) | <0.001 |
| Cardiogenic pulmonary edema | 163 (6.2%) | 66 (9.7%) | 27 (8.7%) | *0.004 |
| Septic shock | 84 (3.2%) | 80 (11.8%) | 35 (11.3%) | *<0.001 |
| Chronic obstructive pulmonary disease | 50(1.9%) | 30 (4.4%) | 17 (5.5%) | *<0.001 |
| Post-operation | 1866(71.4%) | 174 (25.7%) | 54 (17.4%) | <0.001 |
| **Etiology of Respiratory failure** |  |  |  |  |
| Pulmonary system | 415(15.9%) | 259(38.2%) | 149(47.9%) | <0.001 |
| Cardiovascular system | 636(24.3%) | 92(13.6%) | 36(11.6%) | *<0.001 |
| Neurological system | 796(30.5%) | 125(18.4%) | 51(16.4%) | *<0.001 |
| Renal system | 196(7.5%) | 67(9.9%) | 20(6.4%) | 0.075 |
| Gastrointestinal system | 357(13.7%) | 94(13.9%) | 42(13.5%) | 0.984 |
| Other system | 213(8.2%) | 42(6.2%) | 13(4.2%) | 0.016 |
| Ventilation (hours) | 65.5±88.6 | 170.6±108.5 | 325.6±165.1 | <0.001 |
| **Pre-extubation parameter** |  |  |  |  |
| RSI | 49.3±28.3 | 60.4±28.2 | 70.1±39.1 | <0.001 |
| MIP (cmH_2_O) | 38.3 ±14.2 | 36.6±13.1 | 35.6±14.4 | *<0.017 |
| MEP (cmH_2_O) | 63.0±28.5 | 56.2±31.1 | 50.5±28.7 | <0.016 |
| **Weaning methods** |  |  |  |  |
| T-piece | 106(4.1%) | 488 (72.0%) | 266 (85.5%) | <0.001 |
| Pressure support≦8cmH_2_O | 890 (34.1%) | 549 (81.0%) | 237(76.2%) | <0.001 |
| SBT | 994(38.0%) | 661(97.5%) | 307(98.7%) | <0.001 |
| **Number of attempts weaning** |  |  |  |  |
| T-piece | 0±0.2 | 1±0.8 | 3.2±2.4 | <0.001 |
| Pressure support≦8cmH_2_O | 0.3±0.5 | 0.9±0.5 | 0.9±0.7 | *<0.001 |
| SBT | 0.4±0.5 | 1.9±0.7 | 4.1±2.4 | <0.001 |
| **Pre-extubation data** |  |  |  |  |
| FiO_2_ | 27.8±3.8 | 26.6±2.9 | 26.3±2.5 | *<0.001 |
| Pressure level (cmH_2_O) | 9.5±1.6 | 8.4±1.1 | 8.3±1.2 | *<0.001 |
| PEEP (cmH_2_O) | 5.1±0.5 | 5.1±0.5 | 5.1±0.4 | ns |
| Minute ventilation (L/min) | 7.8±2.7 | 7.9±2.4 | 8.0±2.4 | ns |
| Heart rate | 86.9±16.1 | 85.2±17.0 | 87.9±15.9 | ^&^0.041 |
| Mean arterial pressure | 96.8±16.5 | 96.1±15.9 | 93.8±15.2 | ^$^0.006 |
| Respiratory rate | 16.2±5.0 | 17.8±5.3 | 19.5±5.1 | <0.001 |
| pH | 7.435±0.055 | 7.453±0.048 | 7.465±0.044 | <0.05 |
| PaCO_2_ (mmHg) | 36.9±5.8 | 38.9±6.5 | 39.8±6.9 | *<0.003 |
| PaO_2_  (mmHg) | 110.9±45.0 | 93.0±26.8 | 90.9±22.9 | *<0.001 |
| PaO_2_/FiO_2_  (mmHg) | 367.9±103.5 | 339.8±91.5 | 338.8±92.2 | *<0.001 |
| Hemoglobin (g/dL) | 11.6±2.0 | 10.6±1.6 | 10.4±1.5 | *<0.001 |
| Hemotocrit (%) | 34.8±6.5 | 32.4±6.8 | 31.6±6.8 | *<0.001 |
| BUN (mg/dL) | 22.7±18.6 | 30.7±24.8 | 36.9±33.6 | <0.001 |
| Cr (mg/dL) | 1.66±2.09 | 1.77±2.00 | 1.72±2.02 | ns |
| Na (meq/L) | 138.9±4.3 | 139.6±5.3 | 139.7±5.6 | *<0.017 |
| K (meq/L) | 3.8±0.5 | 3.9±0.5 | 4.0±0.5 | <0.022 |
| Ca (mg/dL) | 7.9±1.0 | 7.9±0.8 | 7.9±0.8 | ns |
| P (mg/dL) | 3.4±1.5 | 3.5±1.6 | 3.5±1.5 | ns |
| Albumin (g/dL) | 3.0±0.6 | 2.7±0.6 | 2.5±0.5 | <0.008 |

APACHE-II = Acute Physiology and Chronic Health Evaluation II

TISS = Therapeutic Intervention Scoring System

RSI = Index of Rapid Sallow breathing

MIP = Maximum Inspiratory Pressure

MEP = Maximum Expiratory Pressure

PEEP = Positive end expiratory pressure

SBT = Spontaneous Breathing Trial

* simple versus difficult p < 0.01, simple versus prolonged p < 0.001,

^#^ simple versus difficult p < 0.01

^$^ simple versus prolonged p < 0.01

^&^ difficult versus prolonged p < 0.01
